# *Cyphastrea kausti* sp. n. (Cnidaria, Anthozoa, Scleractinia), a new species of reef coral from the Red Sea

**DOI:** 10.3897/zookeys.496.9433

**Published:** 2015-04-16

**Authors:** Jessica Bouwmeester, Francesca Benzoni, Andrew H. Baird, Michael L. Berumen

**Affiliations:** 1 Red Sea Research Center, King Abdullah University of Science and Technology (KAUST), Thuwal, 23955-6900, Kingdom of Saudi Arabia; 2 Department of Biotechnology and Biosciences, University of Milano-Bicocca, Piazza della Scienza 2, Milan, Italy; 3 ARC Centre of Excellence for Coral Reef Studies, James Cook University, Townsville, QLD 4811, Australia

**Keywords:** Merulinidae, Saudi Arabia, biodiversity, coral reef, taxonomy, KAUST

## Abstract

A new scleractinian coral species, *Cyphastrea kausti* **sp. n.**, is described from 13 specimens from the Red Sea. It is characterised by the presence of eight primary septa, unlike the other species of the genus, which have six, ten or 12 primary septa. The new species has morphological affinities with *Cyphastrea microphthalma*, from which it can be distinguished by the lower number of septa (on average eight instead of ten), and smaller calices and corallites. This species was observed in the northern and central Red Sea and appears to be absent from the southern Red Sea.

## Introduction

The genus *Cyphastrea* Milne Edwards & Haime, 1848 has an Indo-West Pacific distribution range, from the Western Indian Ocean to the Central Pacific, and contains 19 nominal species, nine of which are considered valid (Hoeksema 2014). The genus was previously included in the family Faviidae Gregory, 1900, but recent molecular work has heavily re-organised the classification of the major scleractinian families and *Cyphastrea* is now placed in the Merulinidae Verrill, 1865 (Fukami et al. 2008, Budd et al. 2012, Huang et al. 2014).

In his revision of the genus, based on macro-morphological characters of the skeleton and the tissue of the coral polyps, Matthai (1914) recognised five species, i.e., *Cyphastrea microphthalma* (Lamarck, 1816), *C. serailia* (Forskål, 1775), *C. chalcidicum* (Forskål, 1775), *C. gardineri* (Matthai, 1914), and *C. suvadivae* (Gardiner, 1904). However, the last three were later declared junior synonyms of *C. serailia* by Wells (1954) and Chevalier (1975). Veron et al. (1977) then reinstated *C. chalcidicum* and declared *C. gardineri* as junior synonym of *Cyphastrea microphthalma* rather than *C. serailia*.

More recent regional work on the taxonomy, biodiversity, and distribution of scleractinian corals in the Red Sea originally recognised two *Cyphastrea* species in the region, *Cyphastrea microphthalma* and *C. serailia*, synonymising again *C. chalcidicum* with *C. serailia* (Scheer and Pillai 1983, Sheppard and Sheppard 1991). However, DeVantier et al. (2000) and Veron (2000, 2002) subsequently reinstated *C. chalcidicum* and described a new species from the northern Red Sea, *C. hexasepta* Veron et al. 2000, leading to four valid species of *Cyphastrea* in the Red Sea: *Cyphastrea microphthalma*, *C. serailia*, *C. chalcidicum*, and *C. hexasepta*.

The genus *Cyphastrea* has recently been formally revised within the Merulinidae, incorporating molecular phylogeny, macromorphology, micromorphology, and microstructure (Huang et al. 2014) but it has yet to be revised at the species level. Indeed, the species boundaries within the genus have not yet been investigated using an integrated systematic approach, such as for example in the genera *Plesiastrea* (Benzoni et al. 2011), *Blastomussa* (Benzoni et al. 2014), *Australomussa*, and *Parascolymia* (Arrigoni et al. 2014).

The genus *Cyphastrea* is characterised by its compact coenosteum (Milne Edwards and Haime 1848). The genus is composed of colonial species, with only extracalicular budding. The corallites are monomorphic, monticules are absent, and the coenosteum is generally spinose. Calice width is smaller than 4 mm, septa are in three cycles or less, costosepta are not confluent, and are unequal in relative thickness. The columella is trabecular and compact, and paliform lobes are weak or moderate (Huang et al. 2014). Species in this genus are commonly described based on the septal arrangement (Wijsman-Best 1980, Sheppard and Sheppard 1991, Veron 2000). Among the species currently recognised in the Red Sea, *C. chalcidicum* and *C. serailia* both have two cycles of six septa that are identical in *C. serailia* and alternating in *C. chalcidicum*, *C. hexasepta* has six primary septa, and *Cyphastrea microphthalma* has ten primary septa (Veron 2000). Here we describe a new species that resembles *Cyphastrea microphthalma* in the field but has eight septa: *Cyphastrea kausti*, sp. n. first observed in 2011 in Al Fahal Reef, offshore from the King Abdullah University of Science and Technology, Thuwal, Saudi Arabia.

## Methods

**Figure 1.**
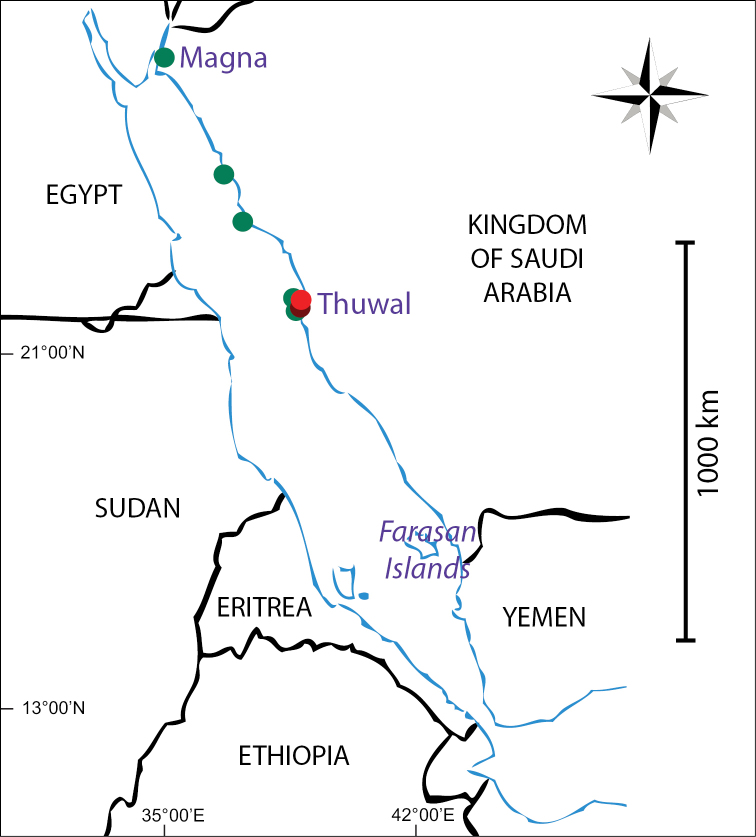
Map of the Red Sea showing the sampling sites of *Cyphastrea kausti* sp. n. The red dot indicates the locality of the holotype, the dark red dot indicates the locality of the paratype, and the green dots indicate the localities of the remaining sampled material.

Colonies of *Cyphastrea kausti* sp. n. (13 in total) were sampled on SCUBA in 2013 during several expeditions along the coast of the Saudi Arabian Red Sea, from Magna in the Gulf of Aqaba in the north to the Farasan Islands in the south (Fig. 1). Digital images of living corals were taken in the field with a Sony DSC-W80 camera and Sony MPK-WB underwater housing and the depth recorded with a dive computer, when possible. Coral specimens were collected with hammer and chisel and tagged. From each colony, a small fragment was subsampled and preserved in absolute ethanol for molecular analysis. The remaining corallum was placed for 24 hours in sodium hypochlorite to remove all coral tissue, rinsed in fresh water, and dried for microscopic observation. The cleaned skeletons were then photographed with a Canon G9 digital camera. Macro and micromorphological characters were examined using light microscopy (Zeiss Stemi 2000 dissecting microscope) and scanning electron microscopy (SEM), respectively. For SEM, a small fragment of clean skeleton was ground at the base with sandpaper, mounted on a stub using double-sided carbon tape, sputter-coated with a 3 nm layer of conductive gold-palladium (AuPd) film, and examined using a Quanta 200 FEG SEM at the KAUST Imaging & Characterization Core Lab. Samples of *Cyphastrea kausti* sp. n. were compared to samples of the other species present in the Red Sea, which were located at the KAUST Biodiversity collection. Specimens of *Cyphastrea kausti* sp. n. were morphologically compared with those of *Cyphastrea microphthalma*, morphologically the most closely resembling species based on *in situ* observations and skeletal examination. The morphometric characters (Table 1) were determined post-imaging with a digital measurement analysis tool calibrated on the image scale bar in Adobe Photoshop CS3. The holotype and a paratype of *Cyphastrea kausti* sp. n. were deposited at the National Museum of Natural History (MNHN), Paris, France. All other material is located at the King Abdullah University of Science and Technology (KAUST), as part of its Red Sea Biodiversity collection. The holotype of *Cyphastrea microphthalma* was examined from images taken by A.F. Budd, available online at http://www.corallosphere.org.

**Table 1.** Micromorphologic characters of *Cyphastrea kausti* sp. n. compared to *Cyphastrea microphthalma*. Average number of septa for seven randomly selected corallites, average calice and corallite width for five randomly selected corallites, and number of corallite centres visible in 1 cm^2^. Standard deviation is indicated for averages.

	*Cyphastrea kausti* sp. n.		*Cyphastrea microphthalma*	
average number septa	MNHN-IK-2012-14236*	8.0 ± 0.3	MNHN-IK-2012-14002*	9.9 ± 0.4
	SA607	8.1 ± 0.4	SA159	9.7 ± 0.8
	SA1121	8.1 ± 0.7	SA552	9.6 ± 0.5
	SA1103	7.7 ± 0.8	SA734	9.9 ± 0.4
			SA100	9.9 ± 0.4
	**Average:**	**8.0 ± 0.4**	**Average:**	**9.8 ± 0.5**
average calice diameter [mm]	MNHN-IK-2012-14236*	1.11 ± 0.06	MNHN-IK-2012-14002*	1.19 ± 0.09
	SA607	1.04 ± 0.03	SA159	1.37 ± 0.05
	SA1121	0.83 ± 0.12	SA552	1.34 ± 0.10
	SA1103	1.05 ± 0.03	SA734	1.28 ± 0.09
			SA100	1.19 ± 0.09
	**Average:**	**1.01 ± 0.13**	**Average:**	**1.27 ± 0.11**
average corallite diameter [mm]	MNHN-IK-2012-14236*	1.77 ± 0.14	MNHN-IK-2012-14002*	1.82 ± 0.12
	SA607	1.61 ± 0.09	SA159	2.14 ± 0.11
	SA1121	1.36 ± 0.11	SA552	2.12 ± 0.17
	SA1103	1.73 ± 0.07	SA734	2.09 ± 0.05
			SA100	1.81 ± 0.08
	**Average:**	**1.62 ± 0.19**	**Average:**	**2.01 ± 0.18**
number corallites per cm^2^	MNHN-IK-2012-14236*	19	MNHN-IK-2012-14002*	33
	SA607	11	SA159	19
	SA1121	31	SA552	11
	SA1103	19	SA734	11
			SA100	19
	**Average:**	**20 ± 8.2**	**Average:**	**15 ± 4.6**

* holotype

### Abbreviations

KAUST King Abdullah University of Science and Technology, Thuwal, Saudi Arabia

MNHN National Museum of Natural History, Paris, France

## Taxonomic account

### Family Merulinidae Verrill, 1865

#### Genus Cyphastrea

Taxon classificationAnimaliaScleractiniaMerulinidae

Milne Edwards & Haime, 1848

Astrea (pars) Lamarck, 1816, not *Astrea* Lamarck, 1801
Cyphastrea Milne Edwards & Haime, 1848
Solenastrea (pars) Milne Edwards & Haime, 1850, not *Solenastrea* Milne Edwards & Haime, 1848

##### Type species.

*Astrea microphthalma* Lamarck, 1816; original designation, Milne Edwards & Haime, 1848

#### Cyphastrea kausti

Taxon classificationAnimaliaScleractiniaMerulinidae

Bouwmeester & Benzoni sp. n.

http://zoobank.org/39E6E02E-B176-4ADB-9175-0E8C29C8D74C

**Figure 2.**
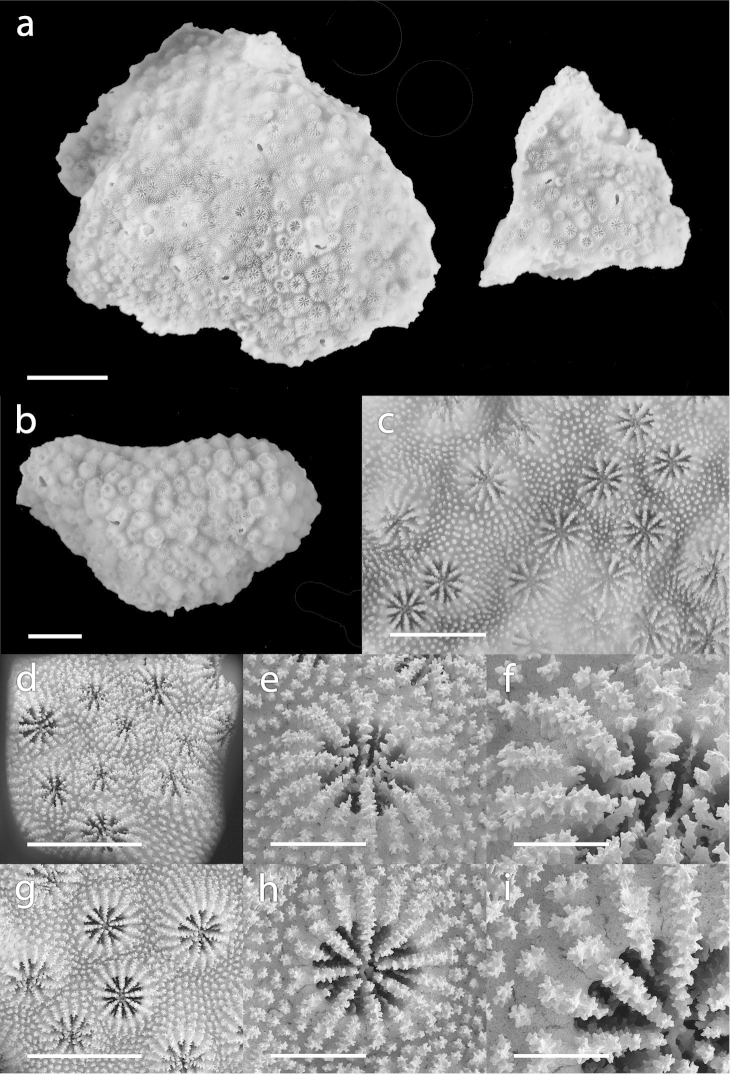
*Cyphastrea kausti* sp. n. **a** holotype, two fragments (MNHN-IK-2012-14236) **b** paratype (MNHN-IK-2012-14237) **c** skeletal detail of holotype (MNHN-IK-2012-14236) **d–f** SEM images of SA1121 **g–i** SEM images of SA1103. Scale bars: 10 mm (**a, b**); 3 mm (**c, d, g**); 1 mm (**e, h**); and 500 µm (**f, i**).

**Figure 3.**
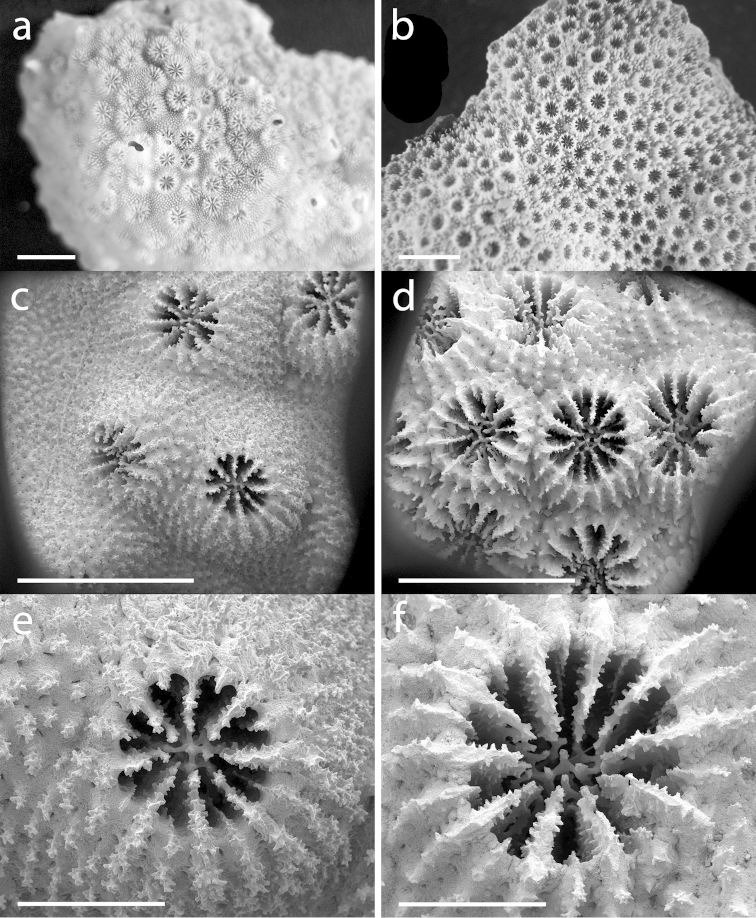
**a, c, e** *Cyphastrea kausti* sp. n. **a** holotype (MNHN-IK-2012-14236) **c, e** SEM images of SA607 **b, d, f** *Cyphastrea microphthalma* **b** holotype (MNHN-IK-2012-14002, photo by AF Budd, with permission from MNHN-Paris) **d, f** SEM images of SA159. Scale bars: 5 mm (**a, b**); 3 mm (**c, d**); 1 mm (**e, f**).

**Figure 4.**
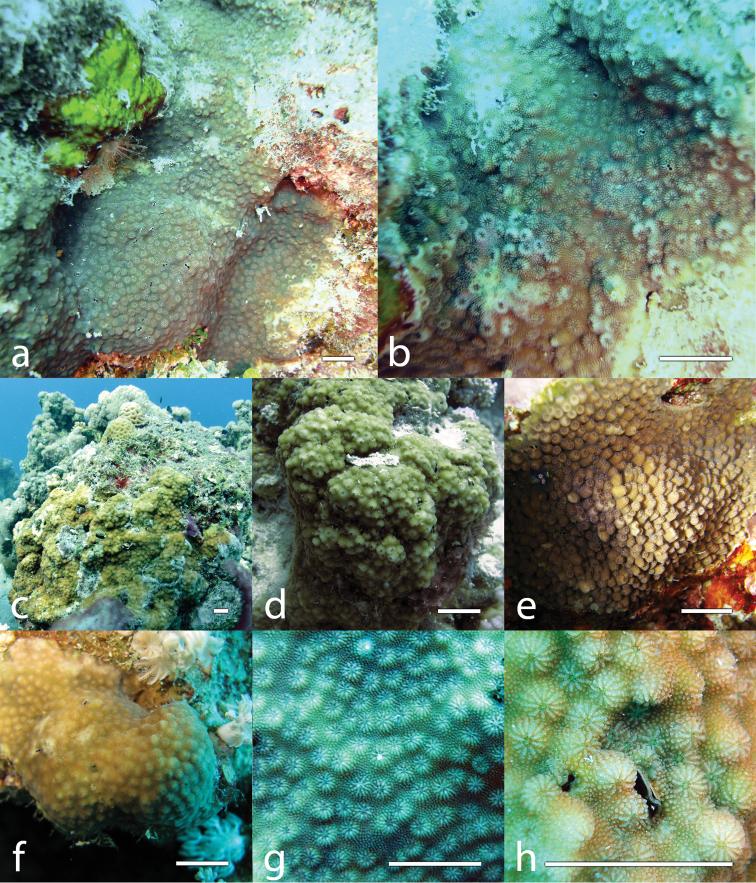
*Cyphastrea kausti* sp. n. **a–b** holotype (MNHN-IK-2012-14236) living colony (Fsar, 13.6 m depth) **c** SAE015 **d** SA498 **e** SA607 **f** SA446 **g** SA644 **h** 7887. Scale bars: 1 cm.

Figures 2 , 3a, c, e , 4

##### Material examined.

**Type material.** *Holotype*: MNHN-IK-2012-14236 (KAUST SA1307). Type locality: Fsar (Thuwal), N 22°13.78', E 39°01.73', depth 13.6 m, coll. J. Bouwmeester 20/10/2013.

*Paratype*: MNHN-IK-2012-14237 (KAUST SA522). Sodfa (Thuwal), N 22°12.07', E 38°57.52', depth 2.0 m, coll. D. Huang, 24/04/2013.

##### Other material (Red Sea, Saudi Arabia).

SA443 Qita al Kirsh (Thuwal), N 22°25.60', E 38°59.77', coll. F. Benzoni, 18/03/2013; SA446 Qita al Kirsh (Thuwal), N 22°25.60', E 38°59.77', coll. F. Benzoni, 18/03/2013; SA498 Sodfa (Thuwal), N 22°12.07', E 38°57.52', depth 10.4 m, coll. D. Huang, 24/04/2013; SA607 Abu Madafi (Thuwal), N 22° 3.73', E 38°45.82', depth 6.1 m, coll. J. Bouwmeester, 28/04/2013; SA643 Tahla (Thuwal), N 22°17.04', E 39° 3.10', depth 6-12 m, coll. J. Bouwmeester, 08/07/2013; SA644 Tahla (Thuwal), N 22°17.04', E 39° 3.10', depth 6-12 m, coll. J. Bouwmeester, 08/07/2013; SA973 Magna (Gulf of Aqaba), N 28°24.23', E 34°44.44', coll. F. Benzoni, 29/09/2013; SA1103 Shaybarah (Al Wajh), N 25°21.69', E 36°54.75', coll. F. Benzoni, 03/10/2013; SA1121 Marker 9 (Yanbu), N 24°26.56', E 37°14.86', coll. F. Benzoni, 04/10/2013; SA1165 Marker 9 (Yanbu), N 24°26.56', E 37°14.86', depth 12.5 m, coll. J. Bouwmeester, 04/10/2013; SAE015 Fsar (Thuwal), N 22°13.78', E 39°01.73', depth 12m, coll. J. Bouwmeester, 21/09/2014.

##### Description of holotype.

The holotype is part of a 12 cm high and 17 cm wide encrusting colony living on an inclined surface (Figure 4a–b), and is constituted of two fragments sampled from a single colony (Figure 2a). The first fragment is the bigger of the two, bell-shaped, 4.5 cm high and 3.8 cm wide (Figure 2a, left). The second fragment is smaller, triangular-shaped, 2.5 cm high with a 2.5 cm base (Figure 2a, right). The number of septa is eight but in a small number of corallites (4/40), the number of septa is seven or nine. The septa are exsert and carry densely ornamented spines. The costae are composed of a line of ornamented spines, which continue on the coenosteum, adding to its already dense and elaborate arrangement of ornamented spines. The columella is trabecular and surrounded by a crown of paliform lobes (Figure 2c). Extra-calicular budding can be observed on both fragments (Figure 2a). The calice diameter of the corallite is 1.11 ± 0.06 mm and the corallite diameter is 1.77 ± 0.14 mm. The corallite density varies from 13 to 22 corallites per cm^2^ (Figure 2c).

##### Diagnosis.

The number of primary septa in *Cyphastrea kausti* sp. n. is typically eight (Figures 2e, h, 3e) but in some cases, seven, nine, or even ten septa can be observed in some corallites of the same colony. However, the majority of corallites have eight primary septa and the average number of septa throughout examined samples is 8.0 ± 0.4 (61 corallites examined from four specimens). This character distinguishes it from *Cyphastrea microphthalma*, which on average has 9.8 ± 0.5 primary septa per corallite, although in one case a corallite with eight septa was observed (Table 1).

In *Cyphastrea kausti* sp. n. the calice diameter of the corallite is 1.01 ± 0.13 mm and the corallite diameter is 1.62 ± 0.19 mm. This is smaller than in *Cyphastrea microphthalma*, which has a calice diameter of 1.27 ± 0.11 mm and a corallite diameter of 2.01 ± 0.18 mm (Table 1).

The corallite density is highly variable between colonies of *Cyphastrea kausti* sp. n. but also within a colony, and varies from 11 to 31 corallites per cm^2^ (e.g. Figures 2c, e, 3c). The corallite density is generally lower on convex surfaces, and higher on concave surfaces, but remains highly variable between colonies, and is not dependant on depth. Indeed, SA607, which had the lowest number of corallites per cm^2^, was sampled at 6 m depth, shallower than the other colonies examined here. In a similar way, corallite density is also highly variable in *Cyphastrea microphthalma* and varies from 11 to 33 corallites per cm^2^.

The first order septa are clearly exsert, the second order septa are weak and never reach the columella, and the third cycle of septa is absent (Figures 2, 3a, c, e). A distinct crown of eight ornamented paliform lobes, corresponding to the number of first order septa, surrounds the columella (Figure 2c). Columella is trabecular and compact (Figures 2h–i, 3c, e). Septal teeth are prominent and ornamented, and granules are scattered on the septal face (Figure 2e–f). Costae are composed of a continuous or dotted line of ornamented spines, which become more and more spaced out while extending on the coenosteum, blending in with the already dense arrangement of ornamented spines, which covers the remaining of the coenosteum (Figure 2c–i). The height of each corallite is also variable, leading to colonies with corallites appearing more exsert than in others (e.g. Figure 4e-f).

##### Field characteristics and identification.

Colonies of *Cyphastrea kausti* sp. n. are encrusting (Figures 4a–c) to submassive (Figure 4d), often growing on inclined substrate, and are found mostly at 6–12 m depth although they have been observed at 2.0–13.6 m depth. They appear similar to *Cyphastrea microphthalma* but close observation of the corallites will reveal the typical eight-arm snowflake septal arrangement (Fig. 2, 3a, c, e). The size of the colonies is variable but generally is 10–60 cm. Colour in the field is cream, yellow, or brown.

##### Etymology.

This species is named after the King Abdullah University of Science and Technology (KAUST), which has facilitated a considerable increase in marine biodiversity research in the Red Sea since its opening in 2009. Moreover, *Cyphastrea kausti* sp. n. was first observed by the authors on Al Fahal, a reef in front of KAUST, in Thuwal, Saudi Arabia.

##### Distribution.

*Cyphastrea kausti* sp. n. has been recorded in the northern and central Red Sea, from Magna in the Gulf of Aqaba, to Thuwal (Figure 1). It was not found in the southern Red Sea, where a similar sampling effort was made.

### Key to the species of *Cyphastrea* from the Red Sea

The primary septa cycle contains:

**Table:** 

1	Six primary septa	**2**
–	Eight primary septa	***Cyphastrea kausti* sp. n.**
–	Ten primary septa	***Cyphastrea microphthalma***
2	Secondary septa cycle absent	***C. hexasepta***
–	Secondary septa cycle present	**3**
3	Primary and secondary septa alternating	***C. chalcidicum***
–	Primary and secondary septa subequal	***C. serailia***

## Discussion

*Cyphastrea kausti* sp. n. is morphologically closest to *Cyphastrea microphthalma* based on the presence of a primary and a secondary septa cycle, a missing third septa cycle, a crown of paliform lobes surrounding the columella (although generally more distinct in *Cyphastrea kausti* sp. n.), the growth form, the densely ornamented coenosteum, and the density of corallites. The two species can, however, be distinguished by the average number of septa (8.0 ± 0.4 for *Cyphastrea kausti* sp. n. and 9.8 ± 0.5 for *Cyphastrea microphthalma*) and by the average calice and corallite size (Figure 3a–f). The calice and corallite diameter are on average smaller in *Cyphastrea kausti* sp. n. (1.01 ± 0.13 mm and 1.62 ± 0.19 mm) than in *Cyphastrea microphthalma* (1.27 ± 0.11 mm and 2.01 ± 0.18). In their description of *Cyphastrea microphthalma* Sheppard and Sheppard (1991) report that Red Sea specimens have a tendency to contain mostly eight primary septa and that this form could be established as a new species in further work, which is done here. Field observations and sampling performed throughout the entire Saudi Arabian coast of the Red Sea between 2013 and 2014 provide evidence that both *Cyphastrea kausti* sp. n. and *Cyphastrea microphthalma* are present in the Red Sea and co-occurring in some regions, often in similar habitats. However, *Cyphastrea kausti* sp. n. appears to be restricted to the central and northern Red Sea and is not found in the southern half of the Red Sea, while *Cyphastrea microphthalma* extends out of the Red Sea and is distributed throughout the Indo-Pacific Ocean with an eastern range limit in Tahiti, French Polynesia, in the Central-Pacific Ocean (Sheppard and Sheppard 1991).

The simultaneous presence of both male and female gametes in colonies of *Cyphastrea kausti* sp. n. during reproductive surveys shows that it is hermaphroditic and likely to spawn in June in the central Red Sea, along with numerous other species including the congeneric *C. serailia*, and *C. chalcidium*, while *Cyphastrea microphthalma* was observed to spawn in May (Bouwmeester et al. 2015). Further reproductive surveys are, however, necessary to establish if indeed a reproductive barrier is present between *Cyphastrea kausti* sp. n. and *Cyphastrea microphthalma* with each species spawning in a different month, or whether one or both of the two species spawn over two consecutive months, as observed in the region for *Acropora humilis*, *Goniastrea edwardsi*, and *Echinopora hirsutissima*, which released gametes during consecutive months in a given year (Bouwmeester et al. 2015).

A molecular phylogeny of all *Cyphastrea* species remains necessary to test and establish species boundaries within the genus. The phylogenetic position of *Cyphastrea kausti* sp. n. will be investigated in further work, integrating a molecular as well as a macromorphological and a micromorphological approach.

## Conclusion

*Cyphastrea kausti* sp. n. is described from the Saudi Arabian Red Sea based on morphological analyses. The eight-septa arrangement in the first septa cycle distinguishes it from other described species in the genus. *Cyphastrea kausti* sp. n. is further recognized by a crown of paliform lobes around the columella and corallite and calice sizes smaller than in *Cyphastrea microphthalma*, to which it is morphologically closely related.

## Supplementary Material

XML Treatment for Cyphastrea

XML Treatment for Cyphastrea kausti
